# A child with a novel *ACAN* missense variant mimicking a septic arthritis

**DOI:** 10.1186/s13052-019-0719-6

**Published:** 2019-11-20

**Authors:** Angelo Florio, Riccardo Papa, Roberta Caorsi, Alessandro Consolaro, Roberto Gastaldi, Marco Gattorno, Paolo Picco

**Affiliations:** 1 Pediatric Rheumatology Clinic, IRCCS Istituto Giannina Gaslini, Via Gerolamo Gaslini 5, 16147 Genova, Italy; 2 Pediatric Endocrinology Clinic, IRCCS Istituto Giannina Gaslini, Genova, Italy

**Keywords:** Pediatric rheumatology, Arthritis, Osteochondritis dissecans, *ACAN*, Aggrecanopathy

## Abstract

Heterozygous mutations of the *ACAN* gene have been associated with a broad spectrum of non-lethal skeletal dysplasias, called Aggrecanopathies. We report a case of a child with severe inflammatory elbow involvement mimicking septic arthritis who carried the new *ACAN* missense variant c.6970 T > C, p.Trp2324Arg. The comprehensive clinical evaluation of the patient and his family, focused on the associated clinical features (facial dysmorphisms, short stature, brachydactily), led us to suspect a hereditary condition. Our findings suggest that Aggrecanopathies should be considered in children with familial short stature, poor growth spurt and joint involvement.

*Sir,*

Aggrecan is a chondroitin sulphated proteoglycan encoded by the *ACAN* gene with essential structural functions in the extracellular matrix of cartilages [1]. Heterozygous *ACAN* mutations have been associated with a broad spectrum of non-lethal skeletal dysplasias, called Aggrecanopathies, including spondyloepimetaphyseal dysplasia, Kimberly type spondyloepiphyseal dysplasia, autosomal dominant short stature, early onset osteoarthritis and recurrent osteochondritis dissecans (OCD) [2–5].

Here, we present a child with severe inflammatory elbow involvement mimicking septic arthritis who carried a new missense variant of the *ACAN* gene.

A 14-year-old boy developed swelling and pain at the right elbow after a physical effort. Symptoms were not associated to other systemic inflammatory signs and worsened during the following days, leading to a severe joint limitation. Laboratory tests and X-ray of the right elbow were normal. Non steroidal anti-inflammatory drugs (NSAIDs) were administered for a month without any improvement.

The patient was admitted to our Institute after 2 months of symptoms onset. On physical examination, acute arthritis of the right elbow was present: it appeared painful and warm, without local erythema. Laboratory tests showed slight elevation of the acute phase reactants (C reactive protein 1.7 mg/dl, erythrocyte sedimentation rate 26 mm/h). Ultrasound revealed a distension of coronoid and olecranon recess with thickening of the synovial membrane. Arthrocentesis was performed: culture tests and cell count of synovial fluid ruled out a septic arthritis. Elbow magnetic resonance imaging (MRI) revealed a bone fragment detachment from the humeral condyle (Fig. 1a) and diagnosis of OCD was made.

**Fig. 1:**
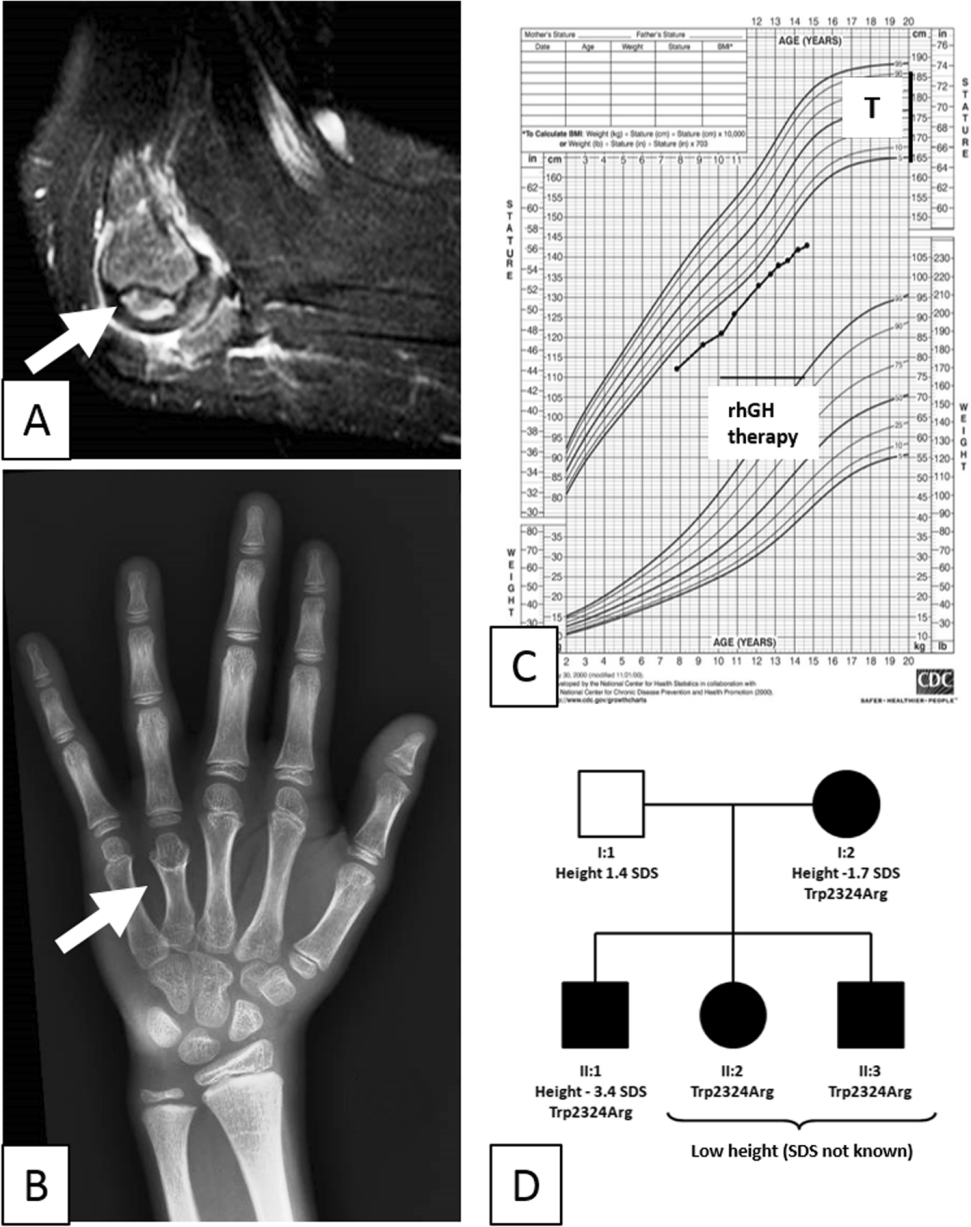
Main features of our patient. Sagittal T2-weighted MRI of the right elbow shows joint distention, synovial thickening, and spongious edema of the humeral condyle with nidus (**a**, arrow tip). X-ray of the left hand shows bone age delay of 2 years with 4th metacarpal bone brachydactyly (**b**, arrow tip). CDC growth chart of the patient (**c**, T = target height). Family pedigree (**d**)

At physical examination, minor skeletal dysmorphisms were noted such as dolichocephaly, hypotelorism, arched palate and brachydactyly of the IV fingers. Moreover, the parents reported a previous episode of OCD at the right knee and pediatric endocrinological evaluations because of severe growth retardation (height always below − 2 SDS).

Notably, when the patient was aging 8 years, short stature was documented (height 112 cm, − 2.4 SDS): insulin tolerance test and L-arginin stimulation test disclosed blunted growth hormone (GH) response (3.16 ng/ml and 14.3 ng/ml maximum peak, respectively). Normal plasma concentration of insulin-like growth factor 1 was detected. Since growth retardation persisted, a further endocrinological evaluation was performed when he was 10 (height 121.4 cm, − 2.5 SDS). X-ray of the left hand revealed a bone age delay of 2 years according the Greulich and Pyle atlas, associated with brachydactyly of the 4th finger (Fig. 1b). Brain MRI showed a partially empty sella without anterior pituitary gland abnormalities. Since the insulin tolerance test showed a hypoglycemic-induced GH peak of 6.27 ng/ml, the diagnosis of isolated GH deficit was pointed out and recombinant human GH (rhGH) treatment started. Nonetheless, poor growth spurt and impaired height velocity rate (− 3.5 SDS) were documented despite the increasing dose of rhGH (Fig. 1c).

The patient was admitted to our Institute with a suspiction of septic arthritis; after the diagnosis of the elbow OCD, we critically re-evaluated the patient history. Namely, i) recurrent episodes of OCD, ii) short stature poorly responsive to the rhGH treatment, and iii) mild skeletal and facial dysmorphisms, led us to hypothesize a form of Aggrecanopathy.

Molecular analysis of the *ACAN* gene by Sanger sequencing revealed the novel missense variant c.6970 T > C, p.Trp2324Arg in the G3 domain of the protein. Intra-familial molecular analysis detected the same variant in the mother, who showed short stature (height 152 cm, − 1.7 SDS), and his two siblings who displayed short stature and brachydactyly, without history of OCD (Fig. 1d).

Since the c.7249G > A variant of the Aggrecan G3 domain has already been described in patients with OCD, short stature, and early-onset osteoarthritis [6], it seems reasonable to hypothesize that the *ACAN* missense variant can explain the overall clinical features of our patient and his family. Moreover, the atypical presentation of our patient highlights a possible role of this new *ACAN* gene variant in joint involvement.

In conclusion, we report a case of Aggrecanopathy where an inflammatory process of the joint mimicking a septic arthritis was the most significant symptom. A comprehensive paediatric evaluation focused on his clinical features (facial dysmorphisms, short stature, and brachydactily) led us to the correct diagnosis and to find other affected family members. Our findings suggest that Aggrecanopathies should be considered in children with familial short stature, poor growth spurt and recurrent inflammatory joint involvement.

## Data Availability

The datasets used and/or analyzed during the current study are available from the corresponding author on reasonable request.

## Abbreviations

GH: Growth hormone
OCD: Osteochondritis dissecans
rhGH: Recombinant human GH
